# Governance Challenges at the Interface of Food Security and Biodiversity Conservation: A Multi-Level Case Study from Ethiopia

**DOI:** 10.1007/s00267-021-01432-7

**Published:** 2021-02-16

**Authors:** Tolera Senbeto Jiren, Julia Leventon, Nicolas W. Jager, Ine Dorresteijn, Jannik Schultner, Feyera Senbeta, Arvid Bergsten, Joern Fischer

**Affiliations:** 1 Faculty of Sustainability, Leuphana University Lueneburg, Lueneburg, Germany; 2 CzechGlobe Global Change Research Institute, Czech Academy of Sciences, Prague, Czech Republic; 3 Carl von Ossietzky Universität Oldenburg, Oldenburg, Germany; 4 Copernicus Institute of Sustainable Development, Utrecht University, Utrecht, The Netherlands; 5 Institute of Development Studies, Addis Ababa University, Addis Ababam, Ethiopia

**Keywords:** Institutional interplay, Institutional structure, Integration, Policy incoherence, Policy output

## Abstract

Ensuring food security while also protecting biodiversity requires a governance system that can address intra- and intersectoral complexity. In this paper, we sought to explore the governance challenges surrounding food security and biodiversity conservation through an empirical study in Jimma zone, southwestern Ethiopia. We used bottom-up snowball sampling to identify stakeholders and then held semi-structured interviews with 177 stakeholders across multiple levels of governance. We also conducted 24 focus group discussions with local people. Data were transcribed and thematically analyzed for its contents. Challenges in the structure of institutions and policy incoherence were the key challenges identified for the governance of food security and biodiversity conservation. The challenges around institutional structure included incompatibilities of the nature of governing institutions with the complexity inherent within and between the two sectors examined. Incoherences in policy goals, instruments, and contradictions of policy output relative to the actual problems of food security and biodiversity further hampered effective governance of food security and biodiversity conservation. Notably, many of the challenges that influenced an individual sector also posed a challenge for the integrated governance of food security and biodiversity conservation, often in a more pronounced way. Based on our findings, we argue that governance in our case study area requires a more integrated and collaborative approach that pays attention to institutional interplay in order to ensure institutional fit and consistency across policy goals.

## Introduction

Simultaneously ensuring food security and biodiversity conservation poses a pivotal challenge for contemporary sustainability governance (Chappell and LaValle 2011). Food security involves the sufficient production and supply of nutritious and culturally preferred food, the physical and economic access to food, and its proper utilization by the population for a healthy and productive life (FAO 2014); whereas biodiversity refers to the variability among all living organisms from all sources; this includes diversity within and between species and their habitats (Convention on Biological Diversity 1992). A key feature of food security and biodiversity governance is that each sector is characterized by the complexity arising from many sub-sectors and policy domains (Chappell and LaValle 2011; Candel 2014), multiple actors with divergent interests and discourses on how to achieve the goals of each sector (Drimie and Ruysenaar 2010; Behnassi and Yaya 2011), and each sector is influenced by many interacting institutional and biophysical factors including multi-scale institutional interplay and power relations (Koc 2013; McKeon 2015).

Public policy in many countries treats food security and biodiversity conservation as separate goals. As a result, interventions target both sectors separately (Glamann et al. 2017), with one sector reinforcing or impeding the success of the other sector (Wittman et al. 2017). For instance, the productivist discourse aims to close yield gaps to improve food security may increase food availability, but it negatively influences biodiversity conservation (Mooney and Hunt 2009). Similarly, approaching biodiversity conservation from a strictly protected area model could enable biodiversity protection, but it can threaten the food security of local people (Naughton-Treves et al. 2005; Fischer et al. 2017). To minimize such trade-offs and improve synergy between the two sectors, integrating these policy sectors appears crucial (Tosun and Leininger 2017).

Increasingly, food security and biodiversity conservation are seen as closely interlinked (Tscharntke et al. 2012), suggesting that these goals can only be sustainably achieved simultaneously (Björklund et al. 2012; Fischer et al. 2017; Torquebiau et al. 2012). Their successful integration, in turn, requires a close fit between the means of governance—the governance system—and the characteristics of particular social-ecological systems (Guerrero et al. 2015). Therefore, strategies are required to govern the food security and biodiversity conservation sectors individually, as well as to address their complex interactions (Björklund et al. 2012; Brussaard et al. 2010; Ericksen et al. 2009).

In this paper, we explore key challenges encountered by governance systems to deliver integrated biodiversity and food security governance. We conceptualize governance as comprising the structures (stakeholders and their linkages), processes (of policy, plans, rules, and their enforcement), and policy content influencing food security and biodiversity conservation (Hill 2013; Mertens et al. 2015). Thus, in the context of this study, governance is understood through the lens of institutions as captured in policies, rules, and plans; as well as the organizations (or stakeholders) established and involved to implement these rules. Such institutions and actors are multilevel, operating across several interdependent tiers (e.g., national, regional, district, kebele, or municipality); and they include a broad range of stakeholders who interplay over multiple jurisdictions (Hooghe and Marks 2001). They are also multi-sectoral, including the policy sectors of food and biodiversity as well as having links with other sectors that influence these. In such complex systems of interacting stakeholders and interests, there are likely to be challenges around institutional interplay, defined as the way in which institutions and stakeholders interact with each other (Young 2002). Issues of interplay need to be considered between institutions on the same governance level (horizontal interplay) as well as between levels (vertical interplay) (Lebel et al. 2005; Paavola et al. 2009; Young 2002). Within each of the horizontal and vertical interplay categories, in situations that bring together multiple interests, there can also be intersectoral interplay between them (Leventon and Laudan 2017).

We focus on a case study in southwestern Ethiopia. Ethiopia hosts a rich but declining biodiversity (Schmitt et al. 2010; Tadesse et al. 2014) and shows high levels of food insecurity (Ethiopian Central Statistical Agency and World Food Programme 2014; Gove et al. 2008). The level of complexity of the governance system for addressing these topics is high, with a large number of stakeholders engaged over multiple governance levels (see e.g., Bergsten et al. 2019; Jiren et al. 2018). Other studies in global south contexts have shown that reduction of food insecurity and biodiversity loss can be hampered by weak institutions (Swiderska et al. 2008), institutional misfits (Bodin and Crona 2009; Brown 2003), divergent sectoral interests (Brown 2003; Guerrero et al. 2015), and a lack of policy coherence (Duit and Galaz 2008). Drawing on our case study, we aimed to: (1) identify problematic vertical interplay within food and biodiversity sector governance; (2) identify problematic horizontal interplay within food and biodiversity governance systems; and (3) identify problematic sectoral interplay between the food and biodiversity governance systems.

Our study contributes to the research on integrated and collaborative governance approaches to social-ecological systems, which—despite criticism regarding practicability and power relations (Bodin 2017)—has been widely regarded as contributing to improved sustainability outcomes (Bodin 2017; Johansson 2018). In particular, we contribute to two main issues that, so far, have remained under-addressed. First, most studies on governance challenges have focused on single sectors (e.g., on food security or biodiversity conservation) or particular governance levels (e.g., municipality or national levels), or a particular aspect of governance (e.g., structures, processes or policies) (Fanning et al. 2007; Sayer et al. 2013). Such fragmented approaches, however, can only partly explain the complex governance challenges emerging from multisectoral and multilevel governance issues (Epstein et al. 2015). Second, despite theoretical progress, detailed empirical studies that holistically assess different aspects of sustainability governance—structures, processes, and policy content—remain scarce (Visseren-Hamakers 2018).

To identify different types of governance challenges, we first introduce a framework of interplay challenges. This framework draws on existing literature to provide a basic classification for the types of governance challenges encountered as a result of horizontal, vertical and sectoral interplay. We then outline our methodology to demonstrate how we applied this framework to our case study in Ethiopia. In the methodology section, we also provide further details of the governance system, including the institutions and actors that we studied. The subsequent results section is structured according to the three objectives in order to demonstrate how vertical, horizontal and sectoral interplay each create governance challenges in our case study. We draw on these results to discuss the implications for improving biodiversity and food security synergies and outcomes in the study area, and for understanding sustainability governance more broadly.

## Conceptual Framework—Identifying the Problematic Interplay

We conceptualized governance challenges as instances where institutional interplay is problematic for the delivery of food security and biodiversity synergies. Such problems foster conflicts, hamper potential synergies and affect the overall effectiveness of governance systems. As explained in the introduction, questions of interplay can be relevant within a single sector (food or biodiversity) or between sectors and can occur vertically between governance levels, or horizontally within a single level. This is summarized in Table 1 as three types of interplay; note that for simplicity, we do not consider vertical, between-sector interplay in our framework because most vertical relationships are among same-sector institutions, while most cross-sectoral challenges manifest as issues of horizontal interplay. Problems of institutional interplay refer to issues arising from institutional as well as stakeholder interactions in the process of setting policies, plans and strategies, implementation and evaluation (Gehring and Oberthür 2008; Young 2002), aiming at the delivery of specific, defined governance outcomes (Paavola et al. 2009; Young 2002). Such challenges are discussed in the literature on environmental governance under various labels (see e.g., Visseren-Hamakers 2018) and focus on various levels of institutional structures and stages of policymaking (Nilsson et al. 2012). Here, we refer to whether they are (i) about institutional structure (the roles and responsibilities of stakeholders); or alternatively (ii) about written policy outputs and instruments.

**Table 1:** Conceptual framework of six types of governance challenges

	Sectoral dimension	
Administrative dimension	Internal (*within* food security or biodiversity)	External (*between* food security and biodiversity)
Horizontal (*within* policy level or implementation level)	A1—Institutional structure A2—Policy output	C1—Institutional structure C2—Policy output
Vertical (*between* policy level and implementation level)	B1—Institutional structure B2—Policy output	N/A

In terms of institutional structure, we focus on the (mis)alignment of institutional and stakeholders’ functions with other institutions and stakeholders, and with the social and ecological characteristics of the system at hand (Duit and Galaz 2008; Folke et al. 2007; Young 2002). We examine whether stakeholders have conflicting or synergistic roles, responsibilities and powers with others. We also consider whether institutions and actors are capable and mandated to govern the functional interlinkages between food security and biodiversity. Notably, we addressed the specific links between food security and biodiversity stakeholders using network analysis in previous papers (Jiren et al. 2017; Jiren et al. 2018; Bergsten et al. 2019). While our past network analyzes yielded useful insights, they did not touch on whether stakeholder interactions were synergistic and constructive, or conflictual and negative to achieving positive outcomes. In contrast to the previous work, here we focus specifically on whether or not the interplay in institutional structure hinders synergies between food security and biodiversity outcomes.

In terms of challenges related to policy outputs, we define policy broadly to include strategies, plans, rules, proclamations, and directives relating to the governance of food security and biodiversity. Problematic interplay within policy outputs occurs when actual policy outputs are not compatible with each other or the related implementation practices or are contradictory to the problem the policy seeks to address (Nilsson et al. 2012). We refer to this phenomenon as policy incoherence. We extended existing notions of policy incoherence, conflict and contradiction between policy elements within a given domain (Candel and Biesbroek 2016; Nilsson et al. 2012), and identified policy incoherences between the sectors of food security and biodiversity conservation.

In summary, we thus considered problems of misaligned institutional structure and problems of incoherent policy output; within a given sector and between sectors; and within a given level of governance (horizontally) and across levels of governance (vertically). The resulting matrix of governance challenges thus considered is summarized in Table 1, and forms the basis of our analyzes in the case study.

## Methodology

### Study System

We focused on the governance of food security and biodiversity conservation in Jimma zone, Oromia regional state, southwestern Ethiopia. Jimma zone is located approximately 350 km southwest of Addis Ababa (Finfinne) and exhibits strong interactions of food security and biodiversity conservation (Ango et al. 2014). We used this zone as a way to anchor and narrow the focus of our research so that it was possible to explore the governance system through the five levels of Ethiopian governance: national/ federal, regional (state), zonal, woreda (district), and kebele (municipality) administration. Studying every stakeholder on every level for the whole of Ethiopia would be very difficult, given logistical, financial and time limitations; however, by connecting our analysis to a specific location, we were able to include virtually all relevant institutions and stakeholders who are directly involved in the governance of food security and biodiversity, and actors that were relevant to that location, while covering both sectors (food and biodiversity) across all levels of governance.

Jimma zone is an area where there is a great benefit to finding and governing, synergies for food security and biodiversity. The vast majority of people living within Jimma zone (90% of the 3.1 million inhabitants) are smallholder farmers who produce cereals and pulses as major food crops, and coffee (*Coffea arabica)* and khat (*Catha edulis)* as cash crops (Oromiya Bureau of Finance and Economic Development 2012). Many additional forest-based ecosystem services are also highly relevant for local livelihoods (Ango et al. 2014). In terms of food security, smallholders in Jimma zone are relatively better off than many people in the other drier parts of the country, but many are still moderately food insecure by international standards (Ethiopian Central Statistical Agency and World Food Programme 2014). Given the diversity of its agroecology, Jimma zone is a hotspot of biodiversity—it is the birthplace of coffee (*Coffea arabica*) (Oromiya Bureau of Finance and Economic Development 2012), the last stronghold of Moist evergreen Afromontane forest (Oromiya Bureau of Finance and Economic Development 2012; Schmitt et al. 2010), and rich in flora and fauna (Hylander and Nemomissa 2008). However, driven by population pressure, agricultural land expansion, and challenges related to the governance system, food insecurity and accelerated biodiversity loss have been major problems in the area (Oromiya Bureau of Finance and Economic Development 2012).

For our particular study, we selected six kebeles in three focal woredas, namely Kuda Kufi and Bereha Werango kebeles from Gumay woreda, Kella Hareri, and Borcho Deka kebeles from Gera woreda, and Difo Mani and Gido Beri from Gumay woreda. Together, these locations spanned a large amount of the social-ecological variation in the study area such as forest cover and livelihood strategies. For instance, Kella Hareri and Borcho Deka kebeles have large forest areas and produce coffee, while people in Bereha Werango and Gido Beri kebeles predominantly produce food crops including maize, sorghum, and teff. In summary, our study thus considered the stakeholders related to food security and biodiversity conservation in six kebeles, three woredas, Jimma zone, Oromia Regional State, and the national level. For the sake of analysis, following the main mandate assigned at each of the administration levels, the five administration levels were further classified into two administrative dimensions, namely the policy level and the implementation level. The policy level involves stakeholders at the national and regional (state) levels, whereas the implementation level consists of the zonal, woreda (district), and kebele (municipality) administration levels.

### Data Collection and Analysis

In order to identify problematic interplay, we collected narrative data from actors engaged in the governance of food security and biodiversity conservation, to understand their perceptions of connections and interplay within the system. To that end, we conducted semi-structured interviews with a representative of each stakeholder within the governance system. We used semi-structured interviews because it facilitates gathering in-depth and rich data on the different governance challenges perceived by individual stakeholders across different governance levels (Kvale and Brinkmann 2009; Ritchie et al. 2013). Because the scope of the study transcends two policy sectors, involves broad institutional interplay challenges across multiple governance levels, a semi-structured interview directly with the stakeholders creates an opportunity for probing and contextualization. We identified stakeholders through the snowball sampling technique (after Leventon et al. 2016; Reed et al. 2009). The use of snowball sampling facilitates nearly a complete enumeration of key stakeholders involved in the governance of food security and the biodiversity sector. We started at the very local level (with the community) and drew on existing knowledge and conversations with key informants to identify kebele level actors. In our semi-structured interviews, we asked for further actors that we should contact, being sure to ask at all levels and for both sectors. We first explained the purpose and scope of our study to the kebele administration and development agents, who then helped us to identify kebele level actors. Here and in all our interactions with actors, we clarified that the food security sector concerned all stakeholders and issues regarding production, access, utilization, and stability of access to food. Similarly, we explained that the biodiversity conservation sector involved actors and issues regarding farmland and forest biodiversity. We identified kebele level actors representing different wealth groups (rich and poor) and geography (geographical household clusters). We used existing official data from kebele offices for the classification into rich versus poor because this official classification of wealth includes contextualized livelihood and food security indicators. Official wealth classification in the area is based on an accounting of household assets such as land holdings, annual income, and food security status. Accordingly, we relied on the already existing official wealth classification to address the rich and poor sections of the community at the kebele level. Based on the listing of actors named by kebele level stakeholders, we identified woreda level stakeholders. We followed a similar procedure at all governance levels until no new actors were mentioned. In this way, we built up a near-complete “map” of the network of stakeholders engaged in the system and sought interviews with many of them. We identified a total of 244 stakeholders involved in the governance of food security and biodiversity conservation. Of these 244 stakeholders, 201 of them participated in this study. The remaining stakeholders, many of them multi-national organizations, declined participation citing limited direct involvement in the governance of food security and biodiversity conservation. We conducted 177 semi-structured interviews in total and 24 focus group discussions with members of the community. More information on the full governance network can be found in (Jiren et al. 2018); all interviewed stakeholders are listed in Table S1.

We departed from our strategy of semi-structured interviews in order to also capture the narratives of local people in the study area. They are important actors within the governance system because of their ultimate role in producing and consuming food and managing the landscape. However, our interview strategy would not have allowed us to capture the diverse perspectives of communities. That means a semi-structured interview with only a few key actors would not make us understand in detail the collective opinions of the community about the governance challenges as well as their differences (Goss and Leinbach 1996; Ritchie et al. 2014). We, therefore, conducted 24 focus groups. These focus groups allowed us to explore opinions and narratives presented by groups representing different sections of communities, as outlined in Table S2.

Respondents in both formats (interviews and focus groups) were asked questions around five main themes: (a) their roles and interest in the governance of food security, biodiversity and the intersection of both, (b) challenges associated with the governance of food security, (c) challenge associated with the governance of biodiversity, (d) challenges for the integrated governance of food security and biodiversity, and (e) the institutions with which they interacted in the governance of food security, biodiversity or both, at either the same governance level or with one level higher. Answers to the topic (e) were used for our snowball sample.

Consent was obtained from participants, and ethics approval was granted by Leuphana University, to audio record interviews and discussions. We transcribed the recordings (average duration: ~1 h) and field notes for all stakeholders. We then organized and entered the transcripts into the qualitative data analysis software NVivo version 11, for the coding of transcripts and subsequent qualitative content analysis (Ritchie et al. 2013; Bryman 2015). Here, we deductively created three separate nodes, one each for food security, biodiversity and one for the integrated governance of both. Next, we created sub-nodes under these primary nodes to identify whether challenges related to horizontal or vertical interplay. We then inductively created another layer of sub-sub-nodes based on the transcripts, in which governance challenges were classified into different themes, where we looked for commonalities and differences in the way in which respondents discussed and presented the challenges that they described to us. This inductive analysis allowed us to explore the six types of governance challenges (see Table 1) related to institutional structures and those that related to policy outputs across the types of interplay. To ensure the quality of coding and reliability in the coding process, intracoding agreement was used by repeatedly coding the transcripts by the same one author at different times and subsequently checked by two authors regarding consistency of codes, before final agreement was reached among the team of authors.

## Results

### Challenges of Horizontal Interplay within a Sector (A1 and A2)

#### Institutional structural gaps within sectors (A1)

At the policy and implementation levels, food security stakeholders had multiple overlapping and intersecting mandates, with limited horizontal coordination between them. These actors focused on similar issues, but the overlapping of mandates led to competition among actors, thereby negatively influencing food security governance. This was especially evident since the 2010 institutional reforms of the food security sector. For example, since 2010, the Bureau of agriculture and natural resources—acting at the regional administration level and below—split into five separate institutions, many of which focus on the same aspects of agricultural production within a common jurisdiction. Few respondents associated overlapping of mandates as an indication of policy emphasis—namely improving food production; whereas most respondents’ criticisms indicated that it had resulted in a neglect of non-production aspects of food security such as economic access, utilization, and stability. Also, institutions compete over scarce resources rather than coordinating tasks towards a common goal. In this regard, for example, a respondent from the disaster prevention and preparedness office stated: “*With a strong emphasis on agricultural production, disaster risk management sectors got less attention and couldn’t cope in a timely way with the regular as well as emerging threats from shocks.*” The partial focus on some aspects of food security caused by the overlapping and intersecting mandates of institutions were the main institutional structural gaps identified at all levels of administration.

In the biodiversity sector, our findings revealed that the absence of institutions (“institutional gap”) that match with the multiple dimensions of biodiversity conservation was the main interplay challenge. Whereas at the implementation level, forest biodiversity was the major concern of institutions (e.g., Oromia Forest and Wildlife Enterprise, Bureau of Agriculture and Natural Resources), biodiversity in other land use types (e.g., farmland) remained unattended by the existing institutions. The words of a respondent at the zonal level capture this particular result: “*No proper institutional support was duly provided to biodiversity conservation in general and farmland biodiversity in particular. This gap is wide at the implementation level.*” The lack of proper attention to those aspects of biodiversity that do not generate immediate financial benefit is likely to exacerbate the loss of this biodiversity, including its unsustainable use in the case of forest biodiversity especially.

Common to both food security and biodiversity sectors, conflicting interests between institutions on resource use appeared as a critical interplay challenge. For example, despite serving the same purpose of ensuring food security, the Bureau of Agriculture and Natural Resources and the Bureau of Coffee Development and Marketing compete over the limited farmland to expand cropland or commercial coffee production, respectively. Such competition was mainly triggered by how stakeholders’ performance at each level was evaluated. They are ranked against one another and rewarded based on what each provides, with no attention to the adverse effect each might cause on another, or possible synergies that could arise from coordinated action. This situation thus created a disincentive to fostering horizontal coordination and communication between institutions. In describing the severity of this challenge, one interviewee stated: “*We understand the importance of coordination and are aware of contradictions between different institutions. But we pursue our task since we will be evaluated in terms of our specific task, and there is no point in wasting resources to foster coordination.*” Lack of coordination and increased resource competition among stakeholders of the same and different sectors was caused by the existing evaluation and monitoring mechanism put in place by the overall administration.

#### Policy incoherence within sectors (A2)

Horizontal policy incoherence within each sector was a reflection of the incompatibility between policy implementation strategies and practices aimed to achieve the desired policy goal. Although the goals of food security policy and biodiversity conservation policy were each consistently reflected in the respective documents (for instance, the rural development policy and strategy), incoherence emerged at the level of strategy and means of implementation of the strategies. For instance, some agencies pursued diversified agricultural production, while others prioritized specialized intensive farming, both on the same kebele, targeting the same group of farmers. Similarly, stakeholders that pursued relatively labor-intensive farming techniques coexist in the same area as others that introduced capital intensive, mechanized farming techniques. Although some respondents acknowledged the plurality of techniques, most respondents indicated that pursuing these strategies in the same area with the same clients (farmers) not only contradicted local conditions (e.g., farmers’ capacity) but also left implementation level stakeholders with contradictory options and incentives.

### Challenges of Vertical Interplay within a Sector (B1 and B2)

#### Institutional structural gap within sectors (B1)

In the food security sector, the incompatibility between the functions of institutions and local operational conditions appeared as the main vertical interplay challenge. This was practically reflected in two ways. First, the quality of interventions in the food security sector was characterized by fragmentation, provisional, and even contradictory to the needs and capacities of local people. Here, the main reported criticism deemed that many interventions were either untimely, discontinued or replaced with other forms of intervention without a thorough evaluation of the preceding interventions. Although prominent in the different dimensions of food security, intervention problems appeared particularly frequently in the supply of food production technologies and financial services. In this regard, many of the discussants indicated that intervention in agricultural technologies, such as improved seed varieties or the use of artificial fertilizers, were among the main issues where intervention was fragmented, provisional, and contradictory to local peoples priorities and capacities. A statement from an interviewee from the community summarizes this challenge: “*Interventions for food security are crooked. Regularly, we are forced to adopt different technologies without seeing the feasibility of previous technology. Sometimes, we adopt different incompatible technologies over the same period by multiple institutions, which leaves us vulnerable.*” Second, we found that interventions in the food security sector were deemed as both supply-push and follow a *one size fit for all* philosophy that often clashes with local preferences. For example, this incompatibility could be summarized by the statement from a focus group discussant: “*Financial institutions such as the Credit and Saving Microfinance Institute provide us agricultural loans without collateral. However, even if it works in other parts of the region, we seldom use it because interest payments contradict with our Sharia law [an Islamic religious law recognized by the formal authority of the country] which prohibits us from paying or receiving financial interest.*” Also, the hierarchical vertical accountability channel left farmers with little decision-making powers about their resources and produce, which consequently led to a misfit of institutional interventions with local preferences. For example, this challenge can be captured by the words of a local farmer that explained this with the analogy of “*What comes from above [referring to God as well as the central government], no one dares to refuse or disobey*” In addition to the local people, many implementation level respondents indicated that the incompatibilities of a nationally developed policy, strategies, and plans with the local level resources, interests and priorities are the main challenges related to institutional structure.

In the biodiversity conservation sector, many key stakeholders were found at the policy level, but with limited coverage at the implementation level, which left numerous aspects of biodiversity conservation unattended (see above section on interplay type A1). This was evident from the Institute of Biodiversity—the main biodiversity conservation institution in the country—which was restricted to the policy level, while no specific institution dealt with biodiversity at the implementation level, especially the woreda and kebele administration levels. Other vertical interplay challenges related to the misfit between the federal political systems of administration with the nature of biodiversity. Here, on the one hand, one challenge was related to the absence of clear responsibility demarcation between actors at the different general-purpose political administration levels. For example, within the policy level, the national-based Ethiopian wildlife conservation authority, and the region-based Oromia Forest and Wildlife Enterprise pursued different and conflicting interests in the governance of wildlife. This caused two main problems in the governance of wildlife. First, driven by income-generating purposes, these stakeholders pursued conflicting interests in the governance of wildlife in the area (see section A1). Second, it created challenges to enforcing nationally endorsed rules and regulations because regional states maintain autonomy, which at times was not aligned with the national biodiversity conservation directions. For instance, a respondent from a policy level stakeholder (National administration level) stressed that: “*Illegal wildlife hunting is widespread in the country, partly because the national institutions face challenges to enforcing rules within their concessional at the implementation level. … the regional states developed their own interests and rarely cooperate with us.*” The vertical interplay challenges caused by the federal political system of administration were also indicated as problematic. On the other hand, many of the respondents revealed that the existing decentralized system only decentralized operational tasks to implementation level institutions, while decision-making powers including resource management and fiscal decisions remained with institutions at the policy levels. This, according to respondents, not only delayed decisions related to biodiversity conservation but also created a gap in enforcing the rules including the punishment of illegal actions at the implementation level, especially at the kebele administration level.

#### Policy incoherence within sectors (B2)

In terms of policy incoherence, many of the strategies set by the policy level biodiversity conservation policy face challenges during implementation, mainly due to practices that deviate from the actual policy content. These conflicting strategies manifested at the implementation level especially at the woreda and kebele levels as incoherent sets of measures and incentives, officially giving communities the right to manage the forest and wildlife in coordination with the government agency through participatory forest management (see Ethiopian Forest Policy of 2007 (FDRE 2007)) while leaving them excluded from resource management decisions, benefit sharing and activities in practice. In addition to this inconsistency in practice, other incoherencies were also found vertically on the irregularities between the different policy contents and actual practices. For instance, at the policy level, the national and regional forest policies and their proclamations (Ethiopian Forest Policy of 2007 and Proclamation 542/2007, (FDRE 2007)) endorsed the establishment of participatory forest management, but no participatory forest management had been established within our study area.

Similarly, Ethiopian national forest policy (Proclamation Number 542/2007, sub-article 3 (FDRE 2007)) also endorsed the protection of endangered tree species (e.g., *Podocarpus falcatus* and *Cordia africana*), while several organizations indicated that these tree species were widely harvested and utilized. Incoherencies also extended to gaps in proclamations and directives. For instance, a regional land use proclamation (ORLP 151/2012, sub.21/5) restricted the plantation of *Eucalyptus* trees in the landscape, with details to be determined by further directives. No such directives, however, were produced to specify the conditionality of such plantations at the implementation level. Due to these conflicting provisions and strategies, implementation-level stakeholders faced considerable institutional uncertainty and socio-economic insecurities. Similar incoherencies of proclamations were observed in the biodiversity sector. For instance, the expansion of private forestry was supported by the policy level national as well as the regional forest policy (Ethiopian National Forest Policy of 2007, (FDRE 2007)), while regional proclamations (ORLP 130/1999 and 151/2001) restricted plantations on private farmland.

### Challenges of the Interplay between Sectors (C1 and C2)

#### Institutional structural gap between sectors (C1)

Institutional interplay challenges identified within each of the sectors were strongly influenced by the integrated governance of the two sectors. However, some of the key institutional interplay challenges were predominant in the integrated governance of food security and biodiversity conservation. These challenges were related to the absence of institutional coordination across the sectors. Two of the key horizontal institutional interplay challenges were institutional instability and weak connecting institutions. Institutional instability and the frequent restructuring of mandates, and associated high turnover of personnel made it difficult to form and maintain lasting coordination between institutions in the two sectors. The magnitude of the challenge of institutional instability was captured by a statement from one respondent who stated: “*Owing to the complexity and interdependence of sectors, cooperation of institutions across these sectors and boundaries is essential. However, because of frequent institutional restructuring and instability, forming and maintaining integration is challenging, and as a result, none of the previous integration attempts succeeded.*” Although the challenge of institutional instability was widely raised by the respondents at the implementation level, the challenge was also among the pertinent problems indicated by the majority of stakeholders at the policy level.

In a related way, weak and missing cross-sectoral linking institutions appeared as the stumbling block to the horizontal integration of food security and biodiversity conservation. Despite the presence of linking institutions (e.g., administration office, cabinets, and councils at all administration levels), and cross-sectoral forums (e.g., agricultural development partnership and linkages advisory council, ADPLAC), none of this fostered coordination between the food security and biodiversity sectors. Limited expertise and lack of willingness in these linking institutions were reported as the reason for the failure to foster cross-sectoral links. In this regard, and as a majority of respondents concurred, a respondent at the implementation level described: “*As a political organization, administrative cabinets and councils (at the woreda and kebele levels) have limited expertise that acknowledges and facilitates coordination between institutions. Moreover, these structures rarely understand synergies, nor are they willing to foster coordination.*” The weakness and missing of stakeholders that play the connecting role across the sectors of food security and biodiversity conservation thus hampered the integrated governance of food security and biodiversity conservation.

#### Policy incoherence between sectors (C2)

At the intersection of food security and biodiversity, policy incoherence was largely related to incoherence in policy goals and discourses. Prioritizing individual goals, as opposed to the integration of the two goals, dominated the policy discourse. To this end, two important but opposing discourses were reported by the majority of respondents. These were: the “biodiversity and sustainable development” vs. “growth and local livelihoods” discourses. The former discourse, rooted in ideas of environmental conservation and supported by a national policy document (FDRE 2011; MOFED 2003), argued that “*the basis for sustainable development and ensuring food security relies on the quality of the environment and natural resources we have. Therefore, taking care of biodiversity is a primary goal*” (interview with a senior policymaker at the national level, and concurred by the majority of respondents in the biodiversity sector). In contrast, the latter discourse, which strongly focused on economic growth and was endorsed by another national policy strategy (FDRE 1995), was characterized, for example, by the statement of a Ministry of Agriculture and Natural Resource representative: “*The primary policy objectives of the nation should be to feed the population using all possible means. Biodiversity conservation needs to support food security.*” This incoherence in discourses was reproduced at the implementation level, leading to the two sectors pursuing conflicting goals, and resulting in conflicts in the integrated governance of biodiversity conservation and food security. National wildlife policy (Ethiopian Wildlife Policy and Strategy, Proclamations 471/2005 and 541/2007, (FDRE 2005)) thus restricted wildlife hunting and population management, which had resulted in an increase of wild animal populations in the forest. Expansions in particular of baboon (*Papio anubis*) and warthog (*Phacochoerus africanus*) populations, in turn, then led to increases in crop-raiding and reduced food security among rural communities. The magnitude of this challenge was captured by the statement of a focus group discussant: “*I harvest less than a quarter of what I plant, the rest is damaged by wildlife.*” The problem of human-wildlife conflict, mainly in the form of crop-raiding, was a challenge in all the study kebeles.

We also discovered that other sectors (e.g., land use and tenure) impacted the interplay between the food and biodiversity sectors. For instance, respondents identified that, at the policy level, the Oromia regional proclamation (Oromia Rural Land Proclamation, ORLP 130/2007) granted rural people assurance against eviction or expropriation, while the same proclamation was endorsing forced expropriation. Similarly, based on provisions under two regional proclamations (ORLP 130/2007 and 151/2012), rural people’s tenure over their land was secured, while the same texts endorsed coercive land transfer for “unused” land.

## Discussion

In our study, we identified that sustainable governance of food security, biodiversity and their integration was often constrained by the challenges of horizontal and vertical interplay, within and between the two sectors. Key findings are summarized in Table 2. These interplay challenges are interrelated and affected not only single policy sectors but also, and often more severely so, the integrated governance of food security and biodiversity. In the following sections, we first discuss our key insights about vertical and horizontal interplay challenges with specific attention to the institutional structure and policy output challenges, followed by a discussion of means of overcoming these governance challenges with a focus on the study area and beyond.

**Table 2:** Summary of key governance challenges affecting food security and biodiversity conservation (also see Table 1)

	Within sector	Between sector
Horizontal	A1—Institutional structure Overlapping of mandates leads to competition between organizations (food) No organizations covering some aspects of biodiversity (biodiversity) Conflicting interests of organizations within a single sector (both) A2—Policy output Conflicting techniques and priorities being pursued in the same locations (food)	C1—Institutional structure No institutional coordination between sectors Frequent restructuring and instability Lack of coordinating actors with skills to integrate C2—Policy output Prioritizing individual goals Opposing discourses and experiences of “biodiversity and sustainable development” vs. “growth and local livelihoods” Broader legal system influences relationships
Vertical	B1—Institutional structure Inconsistent and changeable policies (food) Clash between policy intervention and local cultures and preferences (food) Lack of local level presence (biodiversity) Overlapping and conflicting jurisdictions (biodiversity) B2—Policy output Inconsistency between policy measures and legal responsibilities (biodiversity) Inconsistency between policy content and actual practice (biodiversity)	N/A

In terms of challenges arising from the institutional structure, we found common challenges within each sector, between the sectors, and both horizontally and vertically through the governance system. Namely, we found that at some points in the system, there was an institutional gap, while at other points, there was a concentration of many institutions governing particular aspects of policy sectors (e.g., food production), while yet other aspects (e.g., food utilization, farmland biodiversity) lacked institutional support (A1 and A2). For food security governance, this implies that the existing governance and institutions focused on increasing food production as a leading strategy to ensure food security—the productivism discourse (Koc 2013; McKeon 2015), which pays little attention to the different dimensions of food security, including access, distribution, and equity dimensions. Even the existing strategies around the access and equity dimensions of food security in Ethiopia, such as the productive safety net program, received weak institutional attention in the study area, and these interventions were not mentioned by the stakeholders, including poorer members of the community. The limited institutional attention in the study area is partly because such programs dominantly target household-level emergency food assistance to the poorest in the drier parts of the country (Berhane et al. 2017). The partial focus on the productivism discourse by existing institutions not only influences biodiversity conservation (Mooney and Hunt 2009; Chappell and LaValle 2011), but also the distribution and equity dimensions of food security (Shaw 2007). Concerning biodiversity, institutional support was strong on those resources that generate immediate monetary benefits (A1). Consequently, while institutional redundancy causes a conflict of interest among the institutions around monetarily valuable resources (B1), important biodiversity resources such as farmland biodiversity were left largely unaddressed by institutions, thereby potentially exacerbating their degradation.

Similarly, although institutional flexibility consistent with social-ecological system dynamics is likely to be beneficial (Paavola et al. 2009), overly frequent change and institutional instability hampered cross-sectoral interactions in our case (C1). Institutional instability, i.e., both the frequent change in the mandates and forms of institutions and the frequent change in the bureaucracies (C1), renders the maintenance of cross-sector and cross-scale coordination of stakeholders difficult (Peters 1998). This institutional problem has also been found in other sectors, including water governance (Newig et al. 2016) and rural development and conservation (Mikulcak et al. 2013), and is often the result of the overall national governance regime. In a strongly hierarchical governance system such as in Ethiopia (Dejene 2003; Jiren et al. 2018), maintaining a good institutional structural fit for food security and biodiversity is inherently challenging due to a linear command pathway, which may not be able to capture the inherent complexity of the social-ecological system. Achieving individual as well as integrated goals of food security and biodiversity requires, among other things, institutions that foster collective action within and across sectors, promote institutional learning, and share resources (Berkes 2009; Leventon and Antypas 2012; Candel and Biesbroek 2016).

In terms of challenges arising from policy output, we found clashes in priorities, discourses and techniques within sectors, between sectors, vertically and horizontally (A2, B2, C2). Our findings revealed that food security and biodiversity conservation in southwestern Ethiopia were each characterized by contradictions in sector-specific policy instruments and that strong incoherencies existed in policy goals across sectors. More importantly, we indicated that internally, each policy sector struggled with contradictions in the instruments to achieve specific sectoral goals, such as incoherencies in proclamations and rules related to specific land uses. At a more general level, however, contradictions and incoherence were about fundamental conflicts in policy goals and strategies, e.g., conflicting discourses on the sequencing of development versus conservation. Such content-related challenges are widely acknowledged as major obstacles for sustainability governance and can severely hamper the sustainable achievement of development and conservation goals, and more so when it involves interactions among multiple policies (Brown 2003; Kalaba et al. 2014; Nilsson et al. 2012; Zerbe 2005).

Policy incoherence often stems from defects in institutional structure (Orsini et al. 2013; Nilsson et al. 2012) and leads to conflicting institutional goals (Paavola et al. 2009; Nilsson et al. 2012), thereby hampering the achievement of individual and integrated goals. In the context of food security, biodiversity, and their integration, it is important that a comprehensive approach that ensures participation or coordination of actors from multiple sectors and governance levels is used to maintain policy coherence (Paavola et al. 2009; Nilsson et al. 2012). Our framework presents these challenges separately, but it is important to recognize that they are related, and exacerbate each other. Incoherence in policy outputs can at least partly result from defects in governance structure (or vice versa)—for example, a hierarchical structure limits institutional interactions and encourages disciplinary approaches—as well as defects in governance process—such as failure to effectively pursue jointly agreed policy goals (Nilsson et al. 2012; Orsini et al. 2013). In our study, this is shown, for example, by the food and biodiversity sectors embodying different discourses, and thus structuring their activities differently. Consequently, policy incoherence can lead to governance fragmentation, conflicting institutional goals, and process incompatibility. Thus, it is essential to manage conflicts between policies, and maintain synergies not only between policy goals but also between policy instruments within and across sectors. Overcoming these challenges, and finding synergies between food security and biodiversity conservation in Ethiopia will therefore require simultaneous shifting of both the policy goals and the structures embedded within the governance system in order that they are more closely aligned, between and within sectors and levels of governance.

To address this challenge in Ethiopia, collaborative models of governance (Ansell and Gash 2007; Epstein et al. 2015; Johansson 2018) could enhance structural fit (Bodin 2017; Folke et al. 2005), promote the coordination of institutions and participation across interests and governance levels (Berkes and Ross 2013; Candel 2014), lead to improved coordination of institutional actions (Lubell 2004; Guerrero et al. 2015; Brondizio et al. 2016), and hence create greater coherence of policy contents (Nilsson et al. 2012). In our study area, multiple powerful institutions held structurally central positions and thus could improve coordination between institutions. In practice, however, actual coordination often remains insufficient. Reasons for this include power capture by some stakeholders (Jiren et al. 2018). In addition, in a traditional hierarchical governance system such as in Ethiopia (Dejene 2003; Jiren et al. 2018), maintaining a good fit between governance structures and social-ecological systems is inherently challenging due to a linear command pathway against the complexity of the social-ecological system, and limited room for institutional interaction and learning.

The power of our framework of interplay challenges is that it highlights specific types of problems where interventions can be targeted to increase synergies. For the governance of food security and biodiversity, we specifically showed the types of institutional structures and policy outputs challenges within and between the two sectors in a multi-level governance context (Table 2). Among the many benefits, such analyzes highlight places of intervention within and between the sectors such that interventions reduce trade-offs while increasing the harmonious achievement of the two goals. Each of the individual challenges identified is a complex problem. But being able to see that there is a clash between the discourses pursued by each sector (C2) at least allows for conversation and sharing around these discourses. Highlighting that there is a mismatch between the practices pursued by policies and the priorities and cultures of local people (B1) allows for discussion around how they could better match. Such sharing of worldviews would be the first step towards integration and collaboration (see e.g., Ojha et al. 2010; Allen et al. 2011). As recognized elsewhere (e.g., Ribot et al. 2006; Ostrom 2010; Emerson et al. 2012), our results thus encourage the use of participatory and interdisciplinary approaches to governance in social-ecological systems to overcome procedural and policy-related governance challenges. However, our framework adds a useful tool towards this goal by highlighting the points on which discussion, sharing, and solution-finding should be focused. We, therefore, offer our framework as relevant beyond our case study, to highlight the points of problematic interplay affecting multi-sector, multi-level governance systems. Our framework adds nuance and specificity to understanding how the relationships between sectors and levels are creating problems to synergistic or holistic governance.

## Conclusion

Informed by local people to national-level stakeholders, this study identified key governance challenges associated with institutional interplay, mainly in the form of institutional structure and policy outputs in the context of food security and biodiversity conservation in southwestern Ethiopia. The governance system intended to deliver the integrated outcome in interdependent sectors such as food security and biodiversity conservation faces not only the challenges inherent in the individual sectors but also challenges emergent from the integration of the two sectors. Our study highlighted important challenges to the governance of complex and interdependent sustainability issues and indicated the points at which interventions should be targeted to increase synergies. In our case study, we recommend (1) the establishment of a governance structure that fosters interaction among diverse institutions and sectors across multiple layers of governance, and across jurisdictions; (2) that creating these interactions should be a participatory process that enables the sharing of worldviews, priorities and practical realities. Beyond the context of our case study, we suggest that our framework of interplay problems would be a useful tool for highlighting points in multi-sector, multi-level governance systems where integration needs to be strengthened. Depending on the nature of problematic interplay discovered, appropriate interventions can be planned.

## Supplementary information

Supplementary Material
