# Supplementary material for: Governance Challenges at the Interface of Food Security and Biodiversity Conservation: A Multi-Level Case Study from Ethiopia
Source: Environ Manage. 2021 Feb 16;67(4):717–30. doi: 10.1007/s00267-021-01432-7 (PMC7955995; doi:10.1007/s00267-021-01432-7)
Supplement: Supplementary file 1 — Supplementary Material [file 267_2021_1432_MOESM1_ESM.docx]

# Supplementary material

# Journal of Environmental Management

# Governance challenges at the interface of food security and biodiversity conservation: a multi-level case study from Ethiopia

Tolera Senbeto Jiren^1^**^*^** (toles2006@gmail.com), Julia Leventon^1,5^ ([leventon.j@czechglobe.cz](mailto:leventon@leuphana.de)), Nicolas W. Jager^2^ ([nicolas.jager@uni-oldenburg.de](mailto:nicolas.jager@uni-oldenburg.de)), Ine Dorresteijn^3^ ([i.dorresteijn@uu.nl](mailto:i.dorresteijn@uu.nl)), Jannik Schultner^1^ (jschultner@gmail.com), Feyera Senbeta^4^ ([feyeras@yahoo.com](mailto:feyeras@yahoo.com)), Arvid Bergesten^1^, ([arvid.bergsten@gmail.com](mailto:arvid.bergsten@gmail.com)), Joern Fischer^1^ ([Joern.Fischer@uni.leuphana.de](mailto:Joern.Fischer@uni.leuphana.de))

^1^ Faculty of Sustainability, Leuphana University Lueneburg, Germany

^2^ [Carl von Ossietzky Universität Oldenburg](https://scholar.google.de/citations?view_op=view_org&hl=de&org=14149893444061924580), Germany

^3^Copernicus Institute of Sustainable Development, Utrecht University, The Netherlands

^4^Institute of Development Studies, Addis Ababa University, Ethiopia

^5^ CzechGlobe Global Change Research Institute, Czech Academy of Sciences, Czech Republic

**^*^** Corresponding author / address:

Leuphana University Lueneburg, Universitaetsallee 1, 21335 Lueneburg, Germany; phone: +49 4131 6774034, email: toles2006@gmail.com

**Table S1**: A list of all stakeholders who participated in our study (Column 2), their administration level (Column 3) and policy sectors (Column 4). In the fourth column, which shows stakeholders primariy affiliation, “Both” shows that the staleholder participate in both food security and biodiversity sector; “FS” shows that the stakeholder specifically focus on the food security sector; and “BD” shows that the stakeholder primarily focus on biodiversity sector.

| **Sno** | **Full name of stakeholders** | **Administration Name and Level** | **Sector** |
| --- | --- | --- | --- |
|  | Kebele administration ofice | Kuda kufi Kebele | Both |
|  | Education sector office | Kuda kufi Kebele | Both |
|  | Health extension office | Kuda kufi Kebele | FS |
|  | Agricultural sector development agent office | Kuda kufi Kebele | Both |
|  | Jawi multipurpose community cooperative | Kuda kufi Kebele | Both |
|  | Representative of rich people in the kebele | Kuda kufi Kebele | Both |
|  | Representative of poor people in the kebele | Kuda kufi Kebele | Both |
|  | Women and children’s affairs representative | Kuda kufi Kebele | Both |
|  | Kebele administration office | Bereha Werango Kebele | Both |
|  | Education sector office | Bereha Werango Kebele | Both |
|  | Health extension office | Bereha Werango Kebele | Both |
|  | Agricultural sector development agent office | Bereha Werango Kebele | Both |
|  | Kebele administration office | Difo Mani Kebele | Both |
|  | Education sector office | Difo Mani Kebele | Both |
|  | Health extension office | Difo Mani Kebele | Both |
|  | Agricultural sector development agent office | Difo Mani Kebele | Both |
|  | Ealder from the community | Difo Mani Kebele | Both |
|  | Kebele administration office | Gido Beri Kebele | Both |
|  | Education sector office | Gido Beri Kebele | Both |
|  | Health extension office | Gido Beri Kebele | Both |
|  | Agricultural sector development agent office | Gido Beri Kebele | Both |
|  | Women and children’s affairs representative | Gido Beri Kebele | Both |
|  | Kebele administration office | Kella Hareri Kebele | Both |
|  | Education sector office | Kella Hareri Kebele | Both |
|  | Health extension office | Kella Hareri Kebele | Both |
|  | Kebele administration office | Borcho Deka Kebele | Both |
|  | Education sector office | Borcho Deka Kebele | Both |
|  | Health extension office | Borcho Deka Kebele | Both |
|  | Agricultural sector development agent office | Borcho Deka Kebele | Both |
|  | Oba multipurpose cooperative office | Borcho Deka Kebele | Both |
|  | Arga Union | Gomma woreda | FS |
|  | Bureau of agriculture and natural resources | Gumay woreda | Both |
|  | Irrigation development authority | Gumay woreda | Both |
|  | Disaster prevention and preparedness office | Gumay woreda | FS |
|  | Livestock and fish resource development | Gumay woreda | Both |
|  | Cooperative promotion agency | Gumay woreda | Both |
|  | Land administration and environmental protection | Gumay woreda | Both |
|  | Micro and small enterprise development agency | Gumay woreda | Both |
|  | Oromia credit and finance share company | Gumay woreda | FS |
|  | Trade and market development | Gumay woreda | Both |
|  | Finance and economic development | Gumay woreda | Both |
|  | Education sector office | Gumay woreda | Both |
|  | Health sector office | Gumay woreda | FS |
|  | Water, mineral and energy office | Gumay woreda | Both |
|  | Women and children’s affairs | Gumay woreda | Both |
|  | Civil service and good governance office | Gumay woreda | Both |
|  | Youth and sport office | Gumay woreda | Both |
|  | Administration office | Gumay woreda | Both |
|  | Oromia forest and wildlife enterprise | Gumay woreda | BD |
|  | Rural road authority | Gumay woreda | Both |
|  | Revenues and customs authority | Gumay woreda | Both |
|  | Security and administration office | Gumay woreda | Both |
|  | Bureau of agriculture and natural resource management | Gera woreda | Both |
|  | Irrigation development authority | Gera woreda | Both |
|  | Disaster prevention and preparedness office | Gera woreda | FS |
|  | Livestock and fish resource development | Gera woreda | Both |
|  | Cooperative promotion agency | Gera woreda | Both |
|  | Land administration and environmental protection | Gera woreda | Both |
|  | Micro and small enterprise development agency | Gera woreda | Both |
|  | Trade and market development | Gera woreda | Both |
|  | Finance and economic development | Gera woreda | Both |
|  | Education sector office | Gera woreda | Both |
|  | Health sector office | Gera woreda | FS |
|  | Water, mineral and energy office | Gera woreda | Both |
|  | Japan international cooperation agency (JICA) | Gera woreda | FS |
|  | Community forest users group | Gera woreda | Both |
|  | Women and children’s affairs office | Gera woreda | Both |
|  | Civil service and good governance office | Gera woreda | Both |
|  | Youth and sport office | Gera woreda | Both |
|  | Administration office | Gera woreda | Both |
|  | Oromia forest and wildlife enterprise | Gera woreda | BD |
|  | Rural road authority | Gera woreda | Both |
|  | Transport authority | Gera woreda | Both |
|  | Revenues and customs authority | Gera woreda | Both |
|  | Agricultural growth program | Gera woreda | FS |
|  | Security and administration office | Gera woreda | Both |
|  | Bureau of agriculture and natural resource management | Setema woreda | Both |
|  | Irrigation development authority | Setema woreda | Both |
|  | Disaster prevention and preparedness office | Setema woreda | FS |
|  | Livestock and fish resource development | Setema woreda | Both |
|  | Cooperative promotion agency | Setema woreda | Both |
|  | Land administration and environmental protection | Setema woreda | Both |
|  | Micro and small enterprise development agency | Setema woreda | Both |
|  | Trade and market development office | Setema woreda | Both |
|  | Finance and economic development office | Setema woreda | Both |
|  | Education sector office | Setema woreda | Both |
|  | Health sector office | Setema woreda | FS |
|  | Water, mineral and energy office | Setema woreda | Both |
|  | Women and children’s affairs office | Setema woreda | Both |
|  | Civil service and good governance office | Setema woreda | Both |
|  | Youth and sport office | Setema woreda | Both |
|  | Administration office | Setema woreda | Both |
|  | Rural road authority | Setema woreda | Both |
|  | Transport authority | Setema woreda | Both |
|  | Abay tefases project office | Setema woreda | Both |
|  | Oromia forest and wildlife enterprise | Setema woreda | BD |
|  | Revenues and customs authority | Setema woreda | Both |
|  | Police commission | Setema woreda | Both |
|  | Executive cabinet office | Setema woreda | Both |
|  | Executive council office | Setema woreda | Both |
|  | Ruling political party (OPDO) | Setema woreda | Both |
|  | Security and administration office | Setema woreda | Both |
|  | Bureau of agriculture and natural resource management | Jima Zone | Both |
|  | Irrigation development authority | Jima Zone | Both |
|  | Disaster prevention and preparedness office | Jima Zone | FS |
|  | Livestock and fish resource development | Jima Zone | Both |
|  | Cooperative promotion agency | Jima Zone | Both |
|  | Land administration and environmental protection | Jima Zone | Both |
|  | Micro and small enterprise development agency | Jima Zone | Both |
|  | Oromia credit and finance share company | Jima Zone | Both |
|  | Trade and market development office | Jima Zone | Both |
|  | Finance and economic development office | Jima Zone | Both |
|  | Education sector office | Jima Zone | Both |
|  | Health sector office | Jima Zone | FS |
|  | Water, mineral and energy office | Jima Zone | Both |
|  | Women and children’s affairs office | Jima Zone | Both |
|  | Civil service and good governance office | Jima Zone | Both |
|  | Youth and sport office | Jima Zone | Both |
|  | Administration office | Jima Zone | Both |
|  | Investment commission | Jima Zone | Both |
|  | Rural road authority | Jima Zone | Both |
|  | Transport authority | Jima Zone | Both |
|  | Revenues and customs authority | Jima Zone | Both |
|  | Police commission | Jima Zone | Both |
|  | Executive cabinet office | Jima Zone | Both |
|  | Security and administration office | Jima Zone | Both |
|  | Techno serve | Jima Zone | FS |
|  | Sustainable land management/GIZ project | Jima Zone | FS |
|  | Jima University | Jima Zone | Both |
|  | Capacity building for scaling up best practices (CASCAPE) | Jima Zone | FS |
|  | Ethiopian agricultural research institute | Jima Zone | Both |
|  | Oromia forest and wildlife enterprise | Jima Zone | BD |
|  | Water, mineral and energy office | Jima Zone | Both |
|  | Institute of biodiversity conservation Jimma Botanic Garden | Jima Zone | BD |
|  | Agricultural growth program office | Jima Zone | FS |
|  | Agricultural mechanization research center | Jima Zone | FS |
|  | Plan international | Jima Zone | FS |
|  | Bureau of agriculture and natural resource management, extension division | Oromia Region | FS |
|  | Bureau of agriculture and natural resource management, natural resource division | Oromia Region | BD |
|  | Irrigation development authority | Oromia Region | FS |
|  | Disaster prevention and preparedness commission | Oromia Region | FS |
|  | Livestock and fish resource development | Oromia Region | Both |
|  | Land administration and environmental protection | Oromia Region | Both |
|  | Micro and small enterprise development agency | Oromia Region | Both |
|  | Oromia credit and finance share company | Oromia Region | Both |
|  | Trade and market development bureau | Oromia Region | Both |
|  | Finance and economic development bureau | Oromia Region | Both |
|  | Education sector bureau | Oromia Region | Both |
|  | Health sector bureau | Oromia Region | FS |
|  | Women and children’s affairs bureau | Oromia Region | Both |
|  | Civil service and good governance bureau | Oromia Region | Both |
|  | Oromia forest and wildlife enterprise, forest | Oromia Region | BD |
|  | Oromia forest and wildlife enterprise, wildlife | Oromia Region | BD |
|  | Investment commission | Oromia Region | BD |
|  | Rural road authority | Oromia Region | Both |
|  | Transport authority | Oromia Region | Both |
|  | Revenues and customs authority | Oromia Region | Both |
|  | Water, mineral and energy bureau | Oromia Region | Both |
|  | Agricultural growth program | Oromia Region | FS |
|  | Sustainable land management/GIZ project | Oromia Region | FS |
|  | Cooperative promotion agency | Oromia Region | Both |
|  | Oromia agricultural research institute | Oromia Region | Both |
|  | Agricultural mechanization research center | Oromia Region | Both |
|  | Oromia seed enterprise bureau | Oromia Region | FS |
|  | Oromia Coffee Union | Oromia Region | FS |
|  | Agricultural transformation agency | Federal | FS |
|  | Ministry of agriculture and natural resources | Federal | Both |
|  | Ministry of livestock and fisheries production and management | Federal | FS |
|  | Disaster prevention and preparedness commission | Federal | FS |
|  | International livestock institute | Federal | FS |
|  | Ministry of water, mines and energy | Federal | Both |
|  | Ministry of trade | Federal | Both |
|  | Ethiopian institute agricultural research | Federal | Both |
|  | Institute of biodiversity | Federal | BD |
|  | Ministry of forest, environment and climate change | Federal | BD |
|  | Ethiopian wildlife conservation authority | Federal | BD |
|  | Deutsche Gesellschaft Für Internationale Zusammenarbeit (Germen International Development) Agency) | Federal | Both |

**Table S2:** A list of all stakeholders who participated in the focus group discussion (Column 2), composition of participants in the focus group discussion (Column 3), and their respective administration level (Column 4).

| **Focus group** | **Participants** | **Composition** | **Administration level and and location** |
| --- | --- | --- | --- |
|  | Poor community group | 3 men and 2 women | Kuda kufi Kebele |
|  | Rich community grgoup | 5 men | Kuda kufi Kebele |
|  | General community | 5 men and 4 women | Kuda kufi Kebele |
|  | Community network leaders | 3 men leaders of Zone; and 4 men leaders of Gare | Kuda kufi Kebele |
|  | Poor community group | 4 women and 3 men | Bereha Werango Kebele |
|  | Rich community grgoup | 4 men and 1 women | Bereha Werango Kebele |
|  | General community | 5 men and 3 women | Bereha Werango Kebele |
|  | Community network leaders | 2 men leaders of Zone; and 2 men leaders of Gare | Bereha Werango Kebele |
|  | Poor community group | 4 men and 4 women | Difo Mani Kebele |
|  | Rich community grgoup | 4 men | Difo Mani Kebele |
|  | General community | 7 men and 5 women | Difo Mani Kebele |
|  | Community network leaders | 3 men leaders of Zone; and 1 men leaders of Gare | Difo Mani Kebele |
|  | Poor community group | 3 men and 3 women | Gido Beri Kebele |
|  | Rich community grgoup | 3 men | Gido Beri Kebele |
|  | General community | 5 men and 2 women | Gido Beri Kebele |
|  | Community network leaders | 2 men leaders of Zone; and 4 men leaders of Gare | Gido Beri Kebele |
|  | Poor community group | 3 men and 2 women | Kella Hareri Kebele |
|  | Rich community grgoup | 5 men | Kella Hareri Kebele |
|  | General community | 2 men and 5 women | Kella Hareri Kebele |
|  | Community network leaders | 2 men leaders of Zone; and 3 men leaders of Gare | Kella Hareri Kebele |
|  | Poor community group | 5 men and 3 women | Borcho Deka Kebele |
|  | Rich community grgoup | 6 men | Borcho Deka Kebele |
|  | General community | 8 men and 5 women | Borcho Deka Kebele |
|  | Community network leaders | 3 men leaders of Zone; and 3 men leaders of Gare | Borcho Deka Kebele |
